# Characterisation of gastrointestinal helminths and their impact in commercial small-scale chicken flocks in the Mekong Delta of Vietnam

**DOI:** 10.1007/s11250-019-01982-3

**Published:** 2019-07-02

**Authors:** Nguyen T. B. Van, Nguyen V. Cuong, Nguyen T.P. Yen, Nguyen T. H. Nhi, Bach Tuan Kiet, Nguyen V. Hoang, Vo B. Hien, Guy Thwaites, Juan J. Carrique-Mas, Alexis Ribas

**Affiliations:** 1 Oxford University Clinical Research Unit, Hospital for Tropical Diseases, 764, Vo Van Kiet, District 5, Ho Chi Minh City, Vietnam; 2 Sub-Department of Animal Health and Production, 326-328 Dien Bien Phu, Cao Lanh, Dong Thap Vietnam; 3 Nuffield Department of Medicine, Oxford University, Old Road Campus, Headington, Oxford, UK; 4 Department of Zoology and Fisheries, Faculty of Agrobiology, Food and Natural Resources, Czech University of Life Sciences, Prague, Czech Republic

**Keywords:** Chickens, Emerging farming systems, Helminths, Nematodes, Cestodes, Vietnam

## Abstract

Commercial small-scale chicken farms managed as all-in-all-out but operating with low standards of hygiene/biosecurity are increasingly common in Vietnam. These conditions facilitate the transmission of gastrointestinal helminths. However, there are no published data on helminths in these systems. We aimed (1) to determine the prevalence/burden of gastrointestinal helminths in small-scale commercial flocks in the Mekong Delta region and (2) to investigate the association between worm burdens and birds’ weight and disease status. Randomly selected chickens (*n* = 120) from ‘normal’ flocks were investigated at the end of their production cycle (~ 18 weeks), as well as 90 chickens from ‘diseased’ flocks with signs of respiratory and/or severe disease. The gastrointestinal tract of chickens was dissected and all visible helminths were identified and counted. A total of 54.2% and 54.4% normal and diseased chickens contained helminths. Among colonised birds, the diseased ones harboured a higher mass of helminth worms than normal (healthy) birds (3.8 ± SD 8.6 g vs. 1.9 ± SD 6.3 g, respectively). Eight species were identified, including nematodes (*Ascaridia galli*, *Cheilospirura hamulosa* and *Heterakis gallinarum*), cestodes (*Hymenolepis*, *Raillietina cesticillus*, *Raillietina echinobothrida*, *Raillietina tetragona*,) and one trematode (Echinostomatidae). *Heterakis gallinarum* was the most prevalent helminth (43.3% and 42.2% in normal and sick chickens, respectively), followed by *A. galli* (26.7% and 41.1%). Colonised chickens weighed 101.5 g less than non-colonised birds. Colonisation was higher during the rainy months (May–November) for both *H. gallinarum* and *A. galli*. Anthelminthic usage was not associated with reduced helminth burdens. We recommend upgrading cleaning and disinfection and limiting access to ranging areas to control helminths in small-scale chicken flocks.

**Electronic supplementary material:**

The online version of this article (10.1007/s11250-019-01982-3) contains supplementary material, which is available to authorized users.

## Introduction

Gastrointestinal helminth parasites represent a major constraint to the productivity of small-scale and backyard poultry farming worldwide (Swayne 2013). These helminths interfere with the host’s metabolism, resulting in poor feed utilisation, thus impairing growth and productivity (Gauly et al. 2007; Phiri and Phiri 2007). In addition, helminths may increase disease susceptibility and compromise the immunological response to vaccination (Ayudthaya and Sangvaranod 1997; Pleidrup et al. 2014). Furthermore, gastrointestinal helminths can transmit pathogenic agent such as *Histomonas meleagridis*, which causes high morbidity and up to 20% mortality in chicken flocks (McDougald 2005).

The Mekong Delta hosts about a fifth of Vietnam’s poultry population (Anon. 2018), and about half of rural households are engaged in poultry production (Lan Phuong et al. 2015). Most of the chicken production in the area is non-intensive and is based on slow-growing native breeds. This production mostly takes place in mixed-species farms where chickens are raised in backyards, gardens and orchards. However, in recent years, many Mekong Delta farmers have been upgrading from the backyard to confined ‘all-in-all-out’ systems, although based on traditional, slow-growing native breeds. The number of farms raising more than 100 chickens in Vietnam has increased by 41.5% from 2011 to 2016 (Anon. 2018b). However, to date, there are no published studies on gastrointestinal helminths in chickens flocks in Vietnam. Studies from Asia (India and Thailand) on chickens marketed for meat production have evidenced a high (> 70%) prevalence of colonisation with helminths (Yadav and Tandon 1991; Ayudthaya and Sangvaranond 1997; Katoch et al. 2012; Butboonchoo and Wongsawad 2017). However, such studies were based on chickens collected in local markets, and factors that determine the assemblages of their helminth faunas were not investigated. We carried out a field survey aimed at characterising gastrointestinal helminths in healthy flocks representative of small-scale production of the Mekong Delta of Vietnam, as well as in flocks presenting with severe symptoms of the disease to the veterinary authorities. Specific objectives were (1) to determine the prevalence and burden of gastrointestinal helminths in small-scale, confined flocks and (2) to investigate the potential association between the burdens of helminth infection on the birds’ weight and disease status, as well as other farms, husbandry and climatic variables.

## Methods

### Farms and study location

This study was carried out in Dong Thap, a province located in the Mekong Delta region (southwest of Vietnam). The province has a population density of ~ 510 per km^2^ (Anon. 2014) and is dominated by flood plains. Their main agricultural outputs are rice, fruits, fisheries and livestock (ducks, chickens and pigs). The climate in the Mekong Delta is tropical, with an average temperature of 27.8 °C, with little seasonal variation. The total annual rainfall typically ranges from 1400 to 2400 mm, with a rainy season from May to November, accounting for ~ 90% total rainfall. Farms within two districts (Cao Lanh and Thap Muoi) raising between 100 and 2000 birds as single-age flocks randomly selected from the official provincial census were enrolled into a study within the study province (‘normal’ flocks) (Carrique-Mas and Rushton 2017). In addition, a television spot was run on a local (provincial) television asking owners of chicken flocks with signs of respiratory and/or severe disease to contact the provincial veterinary authorities (Sub-Department of Animal Health and Production of Dong Thap, SDAHP-DT) (‘diseased’ flocks). The study was conducted between May 2017 and March 2018.

### Sample and data collection

‘Normal’ flocks were visited at the end of their production cycle (typically three to five months) where one representative chicken was collected to be examined for the presence of helminths. In addition, data on the farmer’s demographic characteristics, the flock and husbandry practices were collected using structured questionnaires. Data collected from flocks included the following: (1) farmer’s demographics (age, gender); (2) highest level of education attained; (3) experience in chicken farming (years); (4) type of chicken house floor; (5) density of chickens in house/pen (chickens/m^2^); (6) presence of other poultry in the farm (other chicken flocks, ducks and Muscovy ducks); (7) season at the time of necropsy (‘rainy’ season, May to November; ‘dry’ season, December to April); (8) number of chicks stocked; (9) age of the flock (in weeks). The following data was only available from normal flocks: (10) anthelmintics used over the production cycle; (11) percent of weeks when farmers reported disease over the life of the flock (any disease, malaise, diarrhoea or respiratory signs); and (12) average weekly mortality over the production cycle. Data were entered into an Access (Microsoft Office) database. From each farm, one (normal flock) or two (diseased flocks) representative chickens were selected to carry out a post-mortem investigation. The study was approved by the People’s Committee of the Province of Dong Thap. All farm visits were carried out by veterinarians affiliated to the SDAH-DT.

### Examination of chickens for the presence of helminths

Chickens were euthanised following humane procedures (Anon. 2013) by a trained veterinarian. The birds were weighed and their gastrointestinal tract (GIT) was examined for the presence of helminths. The GIT of each bird was systematically separated into three parts: (1) gizzard and proventriculus combined, (2) small intestine (duodenum, jejunum and ileum) and (3) caeca. The gizzard and proventriculus were dissected and examined using a binocular microscope. The small intestine was placed on a large Petri dish containing the saline solution. A pair of scissors was used to dissect it and a scalpel to remove all worms seen attached to the mucosal surface; then the small intestine and its contents were further transferred to a large (500 ml) plastic container, filled with tap water and shaken vigorously. These contents were then filtered several times using a sieve (mesh size 75 μm), and emptied onto a Petri dish. The two ceca were dissected in a Petri dish containing saline solution to facilitate detachment of worms from faeces and mucosa. All worms seen were transferred onto 70% ethyl alcohol-containing tubes pre-labelled with the organ of collection and helminth type (nematode, cestode, trematode). All worms were counted and identified using a binocular microscope.

### Helminth species identification and DNA barcoding

Helminths were identified based on their morphological characteristics (Soulsby 1982; Gomes et al. 2004), and a subset of samples was used for molecular confirmation and identification using DNA barcoding (Gasser and Hoste 1995; Ribas et al. 2013). DNA extraction was performed using the DNeasy Blood and Tissue kit (Qiagen, USA). The first and second internal transcribed spacers (ITS1 and ITS2) as well as the 5.8S rDNA gene were amplified. The PCR reaction used a conserved oligonucleotide primers NC5> 5′-GTAGGTGAACCTGCGGAAGGATCATT-3″ (forward) and <NC2 5′-TTAGTTTCTTTTCCTCCGCT-3″ (reverse). The thermal cycling profile included an initial denaturation in 94 °C for 3 min, followed by 40 cycles at 94 °C for 45 s, 53 °C for 30 s and 72 °C for 1 min, and a final extension cycle at 72 °C for 10 min. PCR products were separated on 1.4% agarose gel run in 1% TBE buffer under constant 120 V for 1 h and the gel stained by Nancy-520 and visualised under UV light. The PCR products were amplified and sequenced using the Big Dye Cycle Sequencing kit (Applied Biosystems, USA) on an ABI 3770 automatic sequence. After generating a sequence type, the sample was inferred to species according to the data available on the Basic Local Alignment Search Tool (BLAST) at the National Center for Biotechnology Information (NCBI).

### Statistical analyses

A sample size of 120 normal chickens and 90 diseased chickens was chosen based on an expected prevalence of helminth colonisation of 65% (in normal) and 80% (in diseased) flocks, respectively, estimated with a precision of 0.085 and a 95% confidence level. The *χ*^2^ test was performed to compare the prevalence of helminths (total and by species) between ‘normal’ and ‘diseased’ flocks. The agreement between the presence/absence of different helminth species in the same bird, as well as over several cycles in the same farm, was investigated by calculating the Kappa statistic. We transformed worm counts of each species to ‘mass of helminth worms’ based on the formula:

$$ W=w\times c $$

where *w* = (*L* × *D*^2^)/(1.6 × 10^6^)where *W* is the mass of helminth worms (μg) of each species present in the bird, *w* is the weight of an individual worm, *L* is the helminth body length (μm), *D* is the body diameter (μm) and *c* is the worm count of each species (Andrássy 1956). A summary variable ‘total mass of helminth worms’ was calculated as the sum of *W* values for each species of helminth present in the bird. The data on each species’ length and diameter used in the weight estimations are provided in Table S1. In order to investigate the impact of worm colonisation on body weight, a linear model was built with the coefficient associated with the variable ‘Presence of helminths’, including bird age, weight and status (diseased/healthy). The agreement between the presence of parasite of each species over subsequent cycles of production was investigated by calculating the kappa values. The Bernoulli spatial model was used to identify potential farm clusters of helminth species using SaTScan software (Information Management Services Inc.) using both normal and diseased flocks (Kulldorff 1997). Linear multivariable regression models were built with study variables in order to identify factors associated with the burden of each parasite species. The outcomes modelled were (1) no. of *Ascaridia galli* worms (log), (2) no. of *Heterakis gallinarum* worms and (3) total no. of cestode worms (log). Variables that were significant at *p* < 0.05 level were kept in the final models.

## Results

### Farms and flocks

**Fig. 1:**
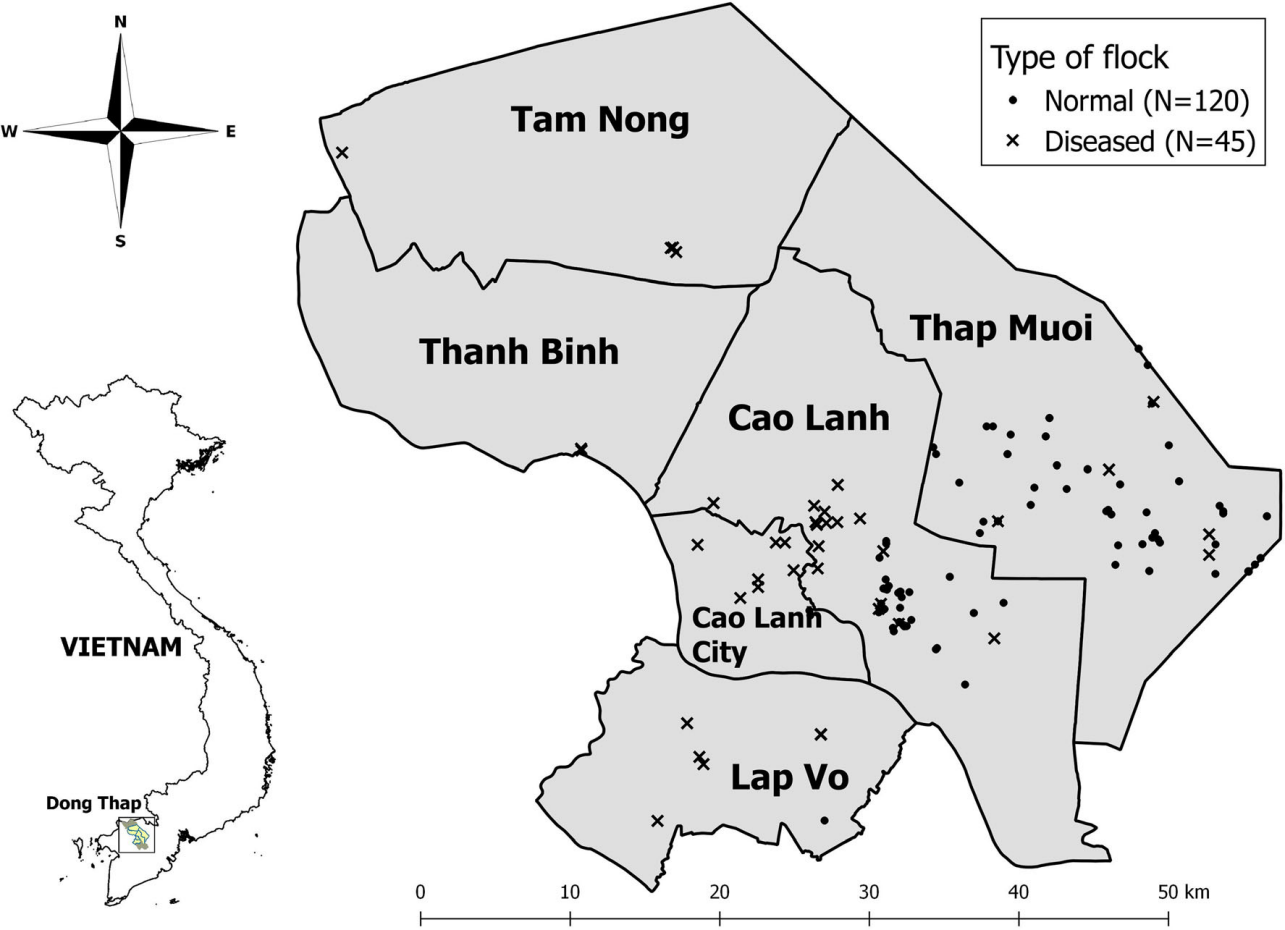
Location of study farms that includes those with ‘normal’ flocks (*N* = 120) and ‘diseased’ flocks (*N* = 45). The names correspond to districts within the Dong Thap province

A total of 120 normal flocks (81 farms, 120 chickens) and 45 ‘diseased’ flocks (45 farms, 90 chickens) were investigated. Diseased flocks were located in a total of five different districts within the province: 49.7% flocks were located in farms in Thap Muoi (district), 37.0% were from farms in Cao Lanh; the rest were from Cao Lanh city (5.5%), Lap Vo (4.2%), Tam Nong (2.4%) and Thanh Binh (1.2%) districts (Fig. 1).

The descriptive features of chicken farms are shown in Table S2. The median size of normal and diseased flocks was 300 [IQR (interquartile range), 200–505] and 165 birds [IQR 100–300], respectively (Kruskal-Wallis *χ*^2^ = 27.047; *p* < 0.001). The median age of chickens of normal and diseased flocks was 18 [IQR 17–20] and 7 weeks [IQR 4–10], respectively (*χ*^2^ = 141.97; *p <* 0.001). The median weight of the chickens investigated was 1800 g [IQR 1500–2200] (normal flocks) and 400 g [IQR 185–632] (diseased flocks) (*χ*^2^ = 150.78; *p* < 0.001). The normal flocks were raised in houses with a soil/earth floor (59.2%), with a cement floor (23.3%), or ‘other’ types floor (wood, sand, metal or a stilted house) (17.5%). Anthelmintics had been used in a third (33.3%) of normal flocks over the production cycle. Data on type of floor and anthelminthic use in diseased flocks were not available.

### Prevalence, burden and distribution of gastrointestinal helminths

Over half of all chickens were colonised by gastrointestinal helminths (65/120 normal birds, 54.2% (95% CI 0.45–0.63); 49/90 diseased birds, 54.4% (95% CI 0.44–0.65). Nematodes were the most common type of helminth (52.5–54.4% birds colonised), followed by cestodes (15.8–16.7%) and trematodes (0–1.0%). A total of 8 different species were identified (Table 1). In healthy birds, the greatest prevalence of colonisation was *Heterakis gallinarum* (43.3%) followed (in decreasing prevalence) by *Ascaridia galli* (26.7%) (nematodes), *Raillietina tetragona* (8.3%), *Raillietina cesticillus* (4.2%) and *Hymenolepis* spp. (2.5%) (cestodes). Among diseased birds, the highest prevalence of colonisation corresponded to *H. gallinarum* (42.2% birds), *A. galli* (41.1%) and *R. tetragona* (11.1%). The prevalence of colonisation with *A. galli* in diseased birds was significantly higher than in healthy birds (*χ*^2^ = 4.231; *p* = 0.04).

**Table 1:** Prevalence (%) of colonisation and worm helminth counts by species. The mean number of helminths and the weight of worms (per bird) correspond to colonised birds

	Normal chickens (*N* = 120)				Diseased chickens (*N* = 90)			
	No. Pos. birds (%)	Total worm count	Mean no. worms (per colonised bird) (± SD)	Mean mass of worms (per colonised bird) (g) (± SD)	No. Pos. birds (%)	Total worm count	Mean no. worms (per colonised bird) (± SD)	Mean mass of worms (per colonised bird) (g) (± SD)
Nematodes	63 (52.5)	2991	47.5 (± 63.4)	0.2 (± 0.4)	49 (54.4)	2135	43.6 (± 107.8)	1.2 (± 4.8)
*A. galli*	32 (26.7)	252	7.9 (± 8.1)	0.4 (± 0.4)	37 (41.1)	1090	29.5 (± 106.1)	1.5 (± 5.5)
*H. gallinarum*	52 (43.3)	2739	52.7 (± 65.4)	0.02 (± 0.02)	38 (42.2)	1042	27.4 (± 33.3)	0.01 (± 0.01)
*C. hamulosa*	0	0	–	–	1 (1.1)	3	3	0.003
Cestodes	19 (15.8)	133	7.4 (± 8.7)	6.0 (± 11.0)	14 (15.6)	405	28.9 ± 78.7	9.0 (± 11.9)
*R. tetragona*	10 (8.3)	71	7.1 (± 9.8)	10.0 (± 13.7)	10 (11.1)	83	8.3 (± 9.4)	11.7 (± 13.2)
*R. cesticillus*	5 (4.2)	11	2.2 (± 1.6)	0.7 (± 0.5)	1 (1.1)	8	8	2.6
*R. echinobothrida*	1 (0.8)	3	3.0	4.2	2 (2.2)	5	2.5 (± 2.1)	3.5 (± 3.0)
*Hymenolepis* spp*.*	3 (2.50)	48	16.0 (± 2.6)	0.02 (± 0.003)	2 (2.2)	309	154.5 ± 205.8	0.17 ± 0.23
Trematodes	0	0	–	–	1 (1.1)	2	2	0.1
Echinostomatidae	0	0	–	–	1 (1.1)	2	2	0.1
Total	65 (54.2)	3124	48.0 (± 64.5)	1.9 (± 6.3)	49 (54.4)	2540	51.9 (± 118.9)	3.8 (± 8.6)

*Pos.* positive, *SD* standard deviation

**Fig. 2:**
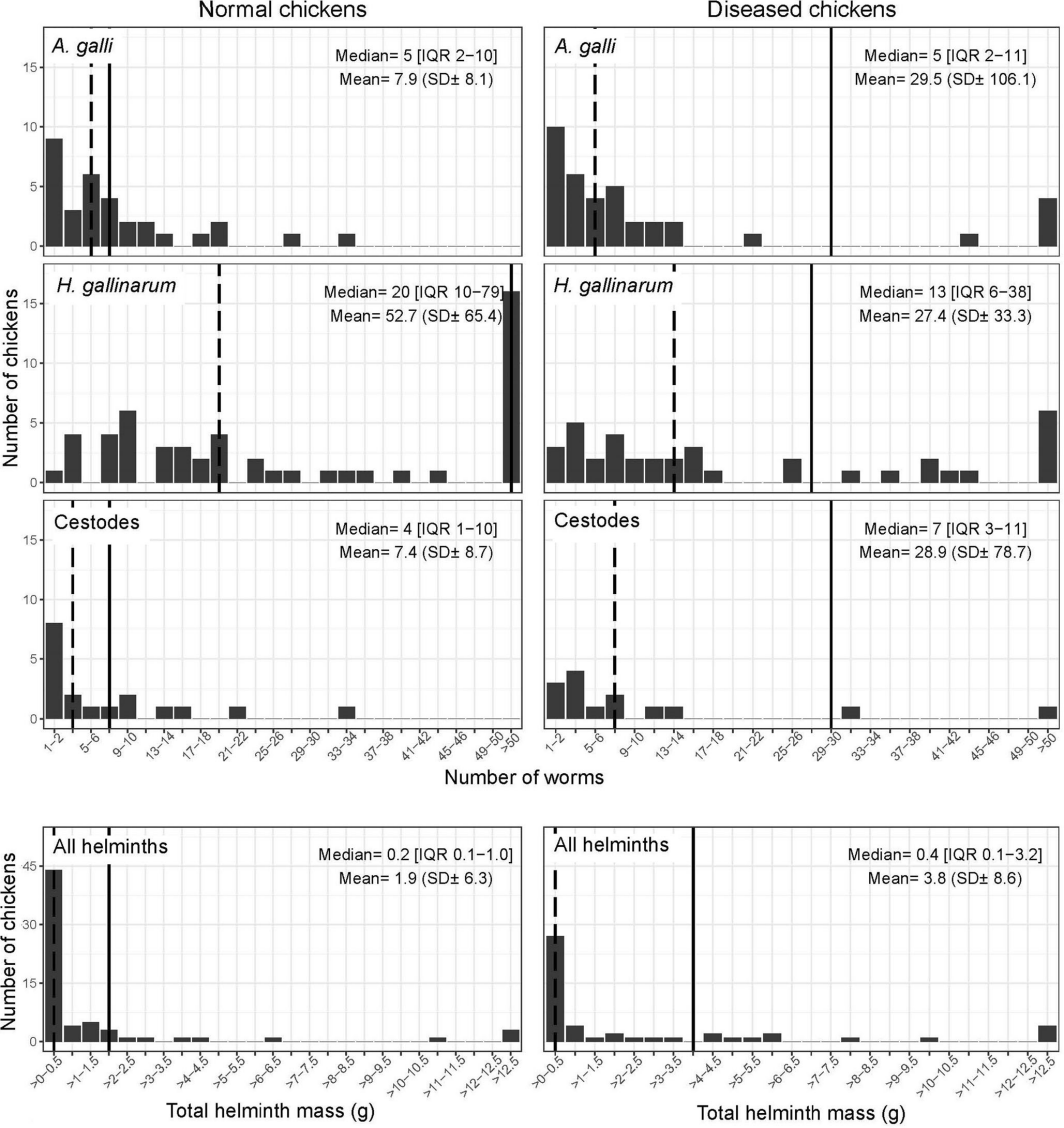
Distribution of counts of worms of each species and total mass of helminth worms among colonised normal and diseased chickens. (Solid line, mean value; broken line, median)

Among colonised birds, those that had clinical signs harboured a higher mass of helminth worms than healthy birds (3.8 ± SD 8.6 g vs. 1.9 ± 6.3 g, respectively). Diseased birds had about a four-fold higher *A. galli* worm count (29.5 ± SD 106.1 worms) compared with healthy birds (7.9 ± SD 8.1 worms). Figure 2 shows the distribution of different helminth species in colonised birds, stratified by disease status. The counts were most skewed for *H. gallinarum* in normal chickens (median 20, mean 52.7). Conversely, the number of *A. galli* worms and mass of helminth worms were also considerably more skewed in diseased birds compared with healthy birds. We found a fair to moderate level of agreement between the presence of *A. galli* and *H. gallinarum* and cestodes, both in diseased and normal chickens (all *p* ≤ 0.001). The highest level of agreement was observed between colonisation with both *A. galli* and *H. gallinarum* in diseased birds (kappa = 0.482; standard error (SE) 0.104) (Table S3).

### Prevalence of gastrointestinal helminths and rainfall

**Fig. 3:**
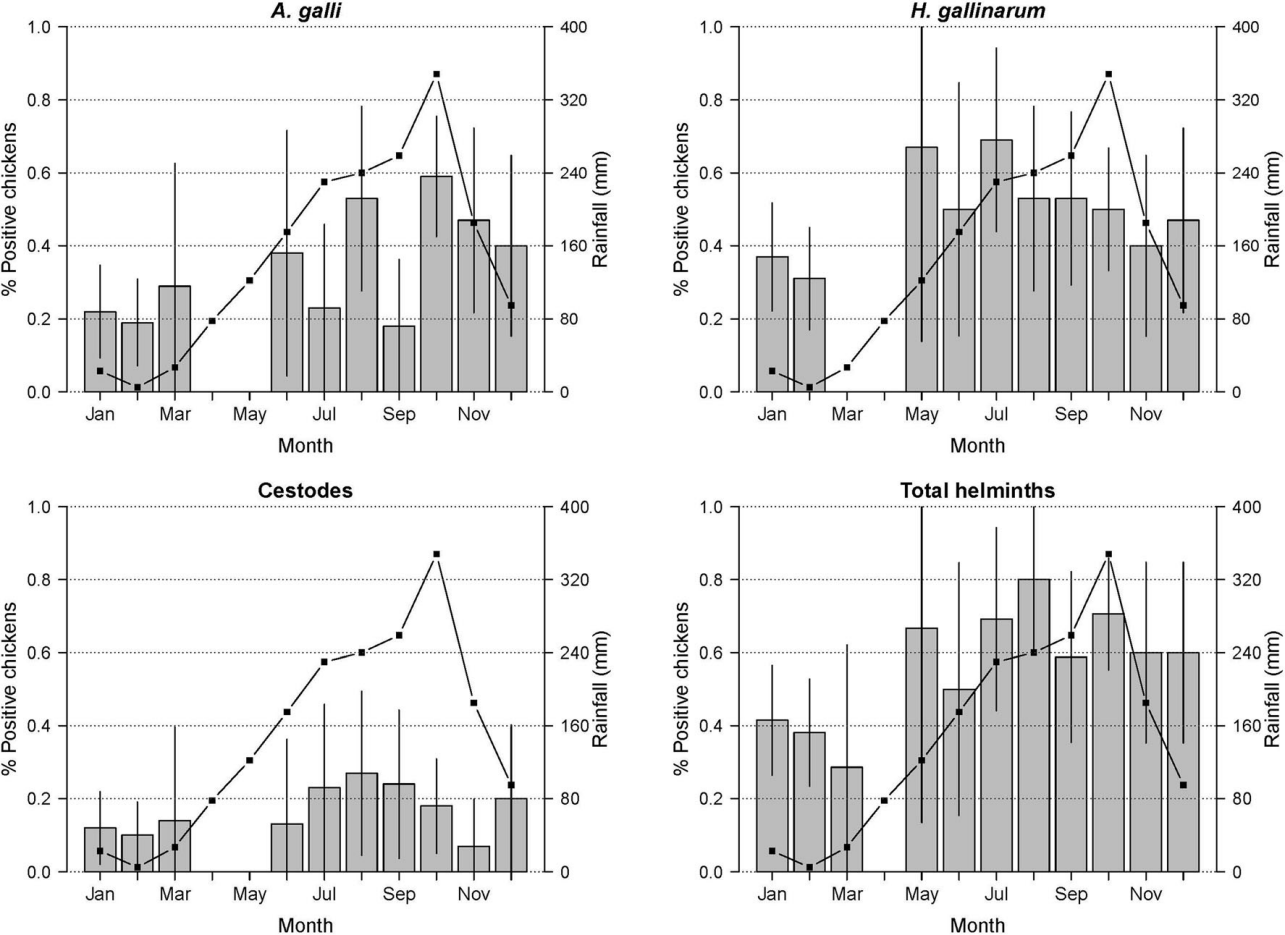
Monthly rainfall (mm) and the proportion of helminth-colonised chickens. Vertical lines indicate 95% confidence intervals around the average prevalence of colonisation by month

The prevalence of chickens colonised with gastrointestinal helminths was 66.7% (95% CI 0.57–0.76) and 41.9% (95% CI 0.32–0.52%) during the rainy and dry season, respectively (*χ*^2^ = 12.0; *p* < 0.001). Similar differences were seen for all three types of helminth (*A. galli*, *H. gallinarum* and cestodes) (Fig. 3).

### Colonisation with helminths over subsequent cycles

In total, there were data for 39 flock transitions observed in 31 normal farms that had two or more cycles. There was no significant correlation between *A. galli* (kappa = − 0.021; standard error (SE) 0.151; *p* = 0.445), *H. gallinarum* (kappa = 0.113; SE 0.129; *p* = 0.189) and cestodes (kappa = − 0.045; SE 0.115; *p* = 0.3488).

### Mass of helminths and chicken body weight

**Fig. 4:**
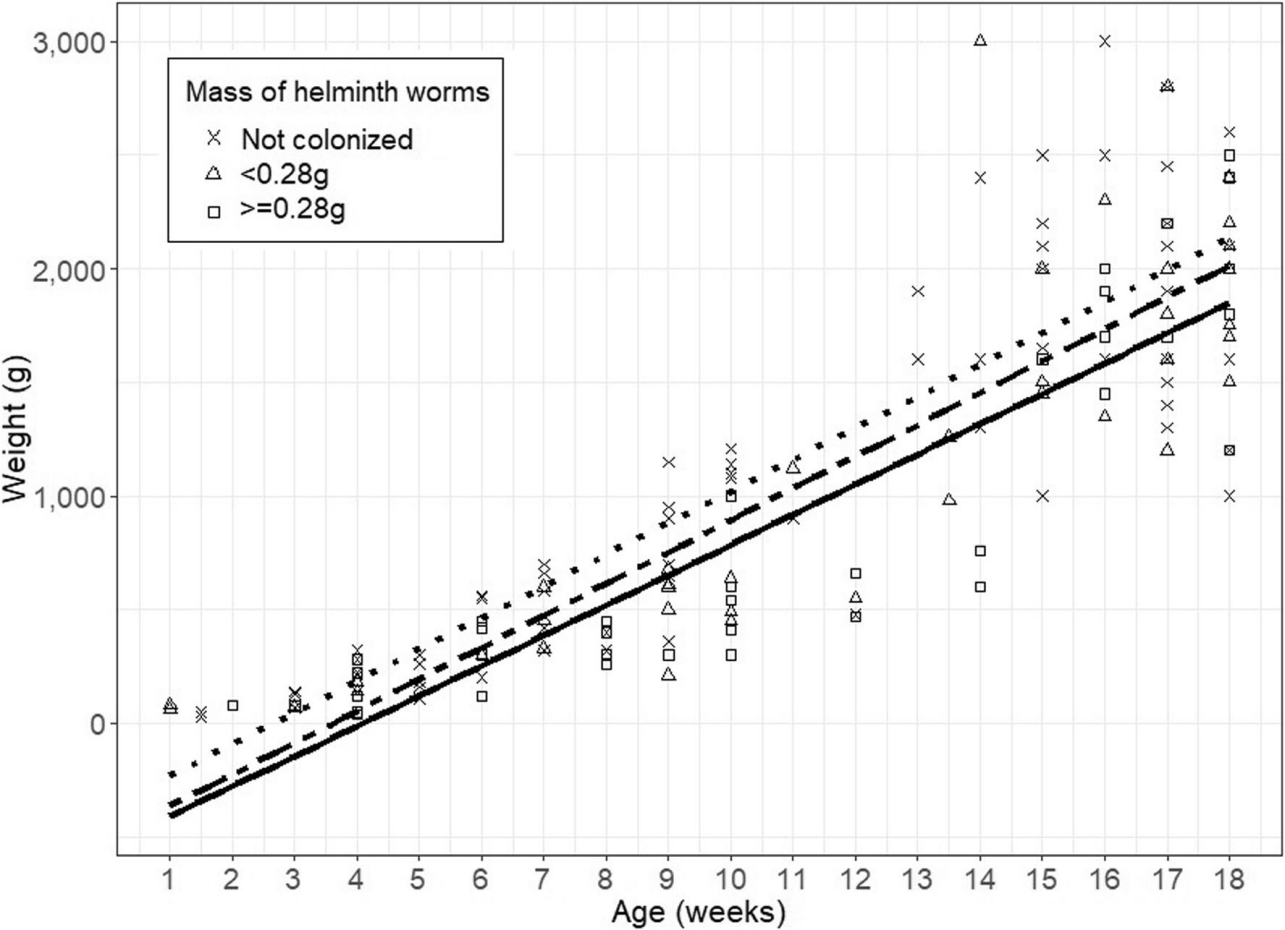
Relationship between the age of chickens (weeks) and their weight (grams), stratified by the severity of helminth colonisation. (Dotted line, not colonised; broken line, colonised with a helminth mass < 0.28 g; solid line, colonised with a helminth mass of ≥ 0.28 g

The relationship between helminth infection status and chicken weight and age is displayed in Fig. 4. The model predicted that colonised chickens weighed 101.5 g (95% CI 0–213.2 g) less than uncolonised chickens.

### Use of anthelmintics

Anthelmintics were used in a total of 40/120 (33.3%) normal flocks. A total of nine farmers had administered anthelminthic to their flocks over the five–week period prior to slaughter, and 31 farmers had administered anthelminthic earlier. A total of seven types of anthelmintics had been used: levamisol (42.5% flocks), followed by menbendazol (20.0%), fenbendazol (17.5%), praziquantel (15.0%), ivermectin (7.5%), sufadimethocine (7.5%) and albendazol (2.5%). Figure S1 shows the timing (week) of administration of anthelmintics in flocks in relation to the mid-point of the cycle.

### Risk factor analyses

Two factors remained significantly associated with the presence of *A. galli* worms: rainy season (*p* = 0.039) and cement floor (compared with soil, stilts and other types of floor) (borderline significant, *p* = 0.089); for *H. gallinarum*: rainy season (*p* < 0.001) and presence of ducks (protective) (*p* = 0.029); for cestodes: cement floor (*p* = 0.009) and presence of ducks (*p* < 0.023) (protective) (Table S4).

### Geographical clustering of helminths

**Fig. 5:**
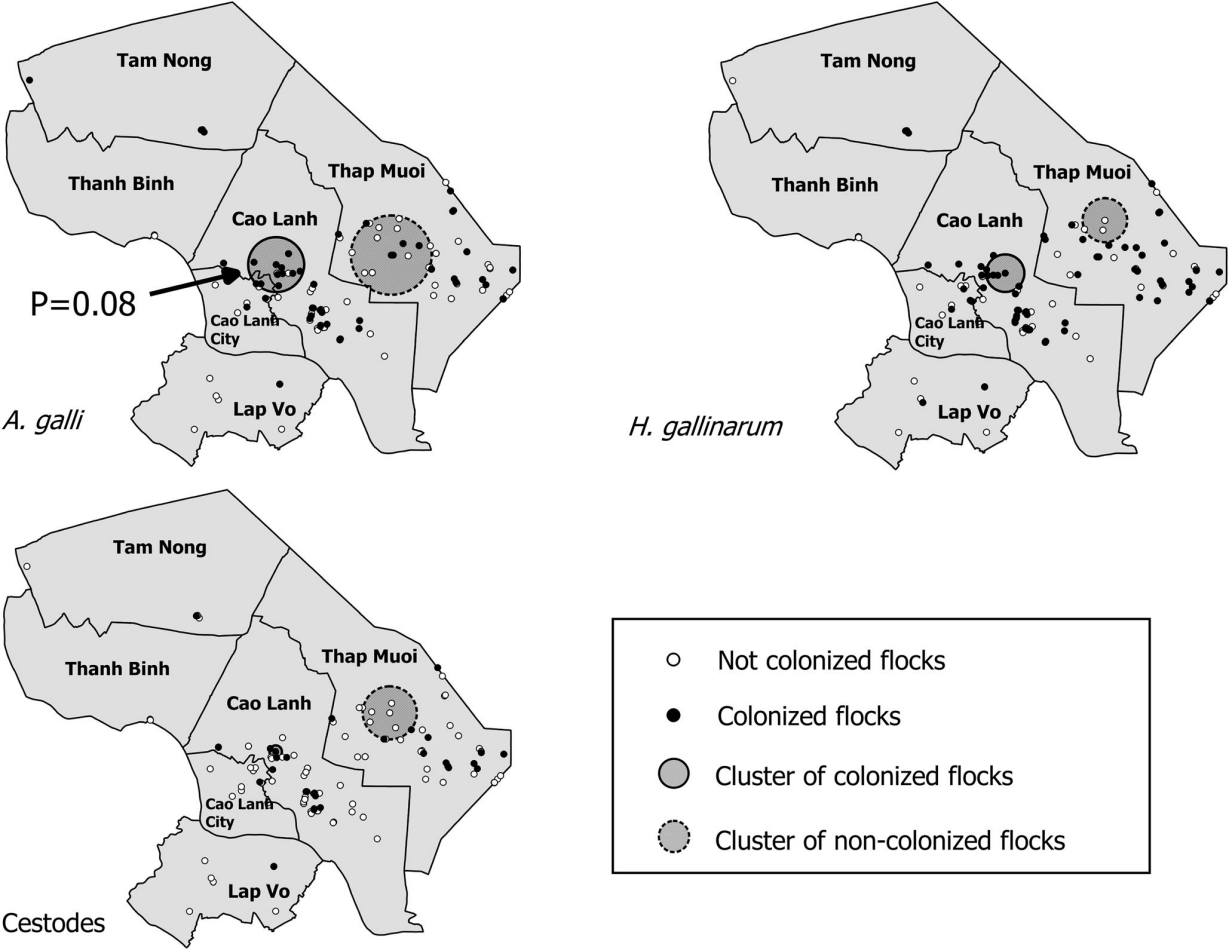
Spatial distribution of flocks colonised and not colonised with *A. galli*, *H. gallinarum* and cestodes in study flocks. The arrow indicates the presence of a significant cluster of colonised flocks at *p* = 0.08

A cluster of the low prevalence of colonisation was found for each of the types of parasites (*A. galli*, *H. gallinarum* and cestodes) in the district of Thap Mui. Clusters of high prevalence of colonisation were also detected for *A. galli* and *H. gallinarum* in Cao Lanh. However, only the cluster in Cao Lanh was significant with *p* < 0.1 (*p* = 0.08) (Fig. 5).

## Discussion

Here, we documented the prevalence, burden and species identity of helminth parasites in healthy and diseased meat chickens raised in commercial, small-scale farms in the Mekong Delta of Vietnam. Our study showed that ~ 54% of chickens in these systems were colonised by gastrointestinal helminths. Gastrointestinal helminths are not an issue in short-cycle chicken flocks (i.e. broilers) raised in modern intensive farming systems; instead, they are still associated with long-cycle birds (i.e. layers) raised in conditions poor biosecurity and deficient hygiene practices (Ybanez et al. 2018; Ola-Fandunsin et al. 2019). Studies on gastrointestinal helminths performed in other countries in Asia show high (> 72%) but variable levels of nematode colonisation in so-called ‘backyard’ and ‘local’ flocks presumably raised for meat at the end of production, with *H. gallinarum* and *A. galli* being the two most common nematode species (Abdelqader et al. 2008, Katoch et al. 2012; Alam et al. 2014; Javaregowda et al. 2016; Butboonchoo and Wongsawad 2017; Wuthijaree et al. 2018). The relatively lower prevalence of colonisation in birds in our ‘normal’ flocks compared with published surveys probably reflects the fact that these flocks were mostly raised in confined conditions. However, sick chickens were younger and generally came from smaller flocks, but had a similar prevalence of colonisation compared with chickens normal flocks. It has been shown that generally older chickens have a higher prevalence of colonisation with helminths (El-Dakhly et al. 2019). Because we did not find overall significant differences in the overall prevalence of colonisation between the two study groups, we hypothesise that this may reflect an interaction between helminths and disease. However, since our diseased flocks tended to be smaller and presumably not raised in conditions of perfect confinement, it is also possible that this reflects differences in the level of challenge. In our study *H. gallinarum* was the most prevalent nematode in non-diseased chickens (43% prevalence) and was found at a higher prevalence than *A galli* (27%). However, in diseased chickens, levels of colonisation with *H. gallinarum* and *A. galli* were comparable in terms of prevalence (41–42%). Given the much higher mass of *A. galli* worms (~ 52 mg) compared with *H. gallinarum* (~ 0.34 mg), this resulted in a considerable overall higher mass of helminth worms among birds colonised with *A. galli*. We also found a significantly higher prevalence of colonisation with *A. galli* among diseased birds. It is likely that conditions in farms that favour transmission of bacterial and viral diseases are also conducive for helminth colonisation. We tested respiratory samples from sick chickens for *Pasteurella haemolytica* by PCR and found a greater detection rate of this bacteria among *A. galli*–positive birds than *A. galli*–negative birds (13.5% vs. 1.8%) (Nguyen T.B. Van, personal communication, 2019). It has been shown that *A. galli* increases the risk of chickens to outbreaks of fowl cholera (Dahl et al. 2002). An additional impact of this parasite is a negative influence on both humoral and cell-mediated immune responses to Newcastle disease vaccination (Pleidrup et al. 2014). However, we do not know the vaccination status of these flocks, even though they all tested negative to Newcastle disease by PCR.

Our normal study flocks (with 100–2000 chickens) are representative of the ‘emerging’ small commercial meat chicken sector in Vietnam. In 2018, only 25% of the ~ 230M of chickens produced annually are raised in large-scale ‘industrial’ farms in this country, with the remaining corresponding to chickens raised in the backyard and small-scale farms (Anon. 2018). Small-scale commercial farms represent an upgrade from backyard production, since flocks are confined and single-age (i.e. ‘all-in-all-out’) and are mostly raised on commercial feed. These production systems are very much on the increase, a phenomenon going in parallel with an unprecedented increase in demand for poultry meat in the country (Anon. 2015).

However, some flocks may at some point be allowed to access a range area (often adjacent to the chicken house). This practice is likely to contribute to the transmission of helminths and may partly explain why ~ 16% healthy flocks were colonised with cestodes. We did not find, however, clear evidence of carry-over over consecutive cycles, suggesting that probably chickens become colonised when accessing random areas outside the chicken pens or due to the supplementation of contaminated plants as feed. Some farmers are known to supplement their flocks with fresh plants, often including aquatic plants which can be contaminated with infective larvae as well as with aquatic snails. The latter may explain the presence of echinstomatids in one flock, since this cestode is related to the ingestion of aquatic snails that act as second intermediate host-harbouring infective metacercariae.

We empirically demonstrated the economic impact of helminth parasites on chicken bodyweight. A study in India compared the body weight of chickens after 3 months that were treated with anthelminthic and compared with a control (untreated group). The observed difference in bodyweight was ~ 385 g (Katoch et al. 2012), which was considerably greater than our study (101.5 g). However, chickens in the Indian study were mostly backyard, and the prevalence of cestodes in that study was much higher.

We found a generally higher prevalence of colonisation of flocks during the rainy season. Climatic conditions such as rainfall and temperature are known to influence the population dynamics of helminths (Mas-Coma et al. 2008). The Mekong Delta is characterised by high temperature and humidity, a climate highly suitable for the development of parasites outside their final host, facilitating egg embryonation (Saunders et al. 2000; Tarbiat et al. 2015), as well as the presence of insect intermediate hosts (in the case of cestodes and trematodes) (Abebe et al. 1997).

A surprising finding was the apparent no differences in colonisation between flocks treated and not treated with anthelmintics. However, most farmers had used anthelmintic drugs only sporadically, and relatively few farms had applied anthelminthic in the latter phase of the production cycle. This lack of effect of anthelminthic use can reflect either inadequate preparation of the products (wrong storage, dilution etc.) or the presence of-of anthelmintic resistance against the products used.

Flocks raised on cement floor had unexpectedly a higher prevalence of colonisation with *A. galli* and cestodes compared with those raised on soil/earth floor (including stilt houses). We cannot explain this finding, since it is generally assumed that an earth floor contributes to maintaining the life cycle of these parasites, since eggs of nematodes can survive for longer periods (Anderson 2000). The presence of ducks was associated with a lower risk of colonisation with *H. gallinarum* and cestodes. A possible explanation for the lower prevalence of cestodes in the presence of ducks is their capacity for predating arthropod vectors.

In summary, we characterised levels and burdens of colonisation with helminths in chickens raised in emerging, commercial small-scale chicken farming systems in the Mekong Delta of Vietnam. We demonstrated that nematodes account for the loss in productivity of such flocks, nematodes being the most abundant type of parasite, and *A. galli* being associated with disease in flocks. We recommend to step up biosecurity and hygiene measures aiming at reducing the prevalence of colonisation with helminths. The observed lack of clear evidence of a protective effect from anthelmintic use warrants further investigation.

## Electronic supplementary material

ESM 1 (DOCX 400 kb)

ESM 2 (DOCX 15 kb)

ESM 3 (DOCX 16 kb)

ESM 4 (DOCX 16 kb)

ESM 5 (DOCX 15 kb)
