# Supplementary material for: Characterisation of gastrointestinal helminths and their impact in commercial small-scale chicken flocks in the Mekong Delta of Vietnam
Source: Trop Anim Health Prod. 2019 Jul 2;52(1):53–62. doi: 10.1007/s11250-019-01982-3 (PMC6969868; doi:10.1007/s11250-019-01982-3)

Figure S1: Timing of administration of anthelminthics in 120 normal flocks by week in relation to the mid-point of production. Flocks are sorted by their cycle duration. Grey=Not used anthelminthic. Black=Used anthelminthic.


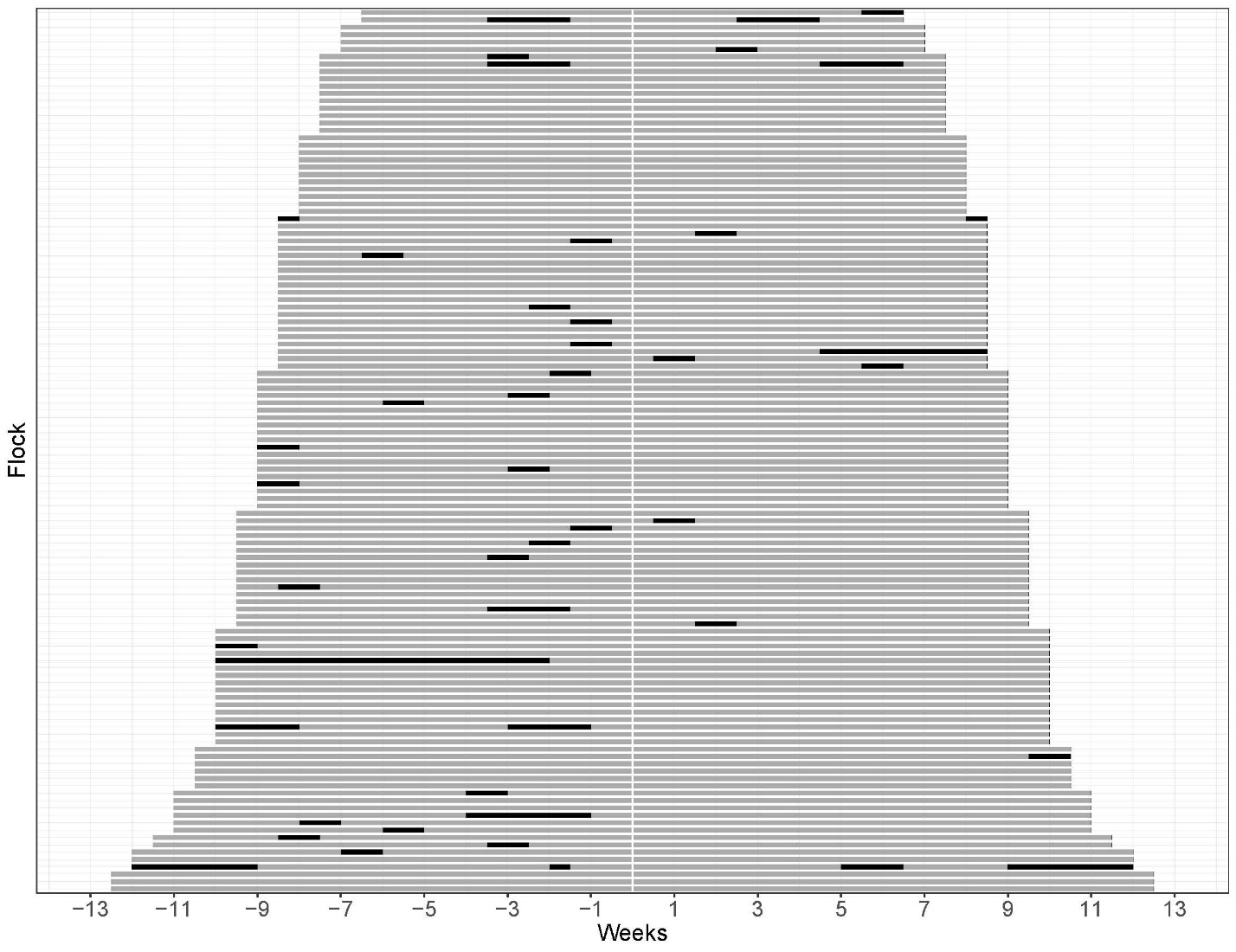

Supplement: Supplementary file 1 — (DOCX 400 kb) [file 11250_2019_1982_MOESM1_ESM.docx]
