# Supplementary material for: Characterisation of gastrointestinal helminths and their impact in commercial small-scale chicken flocks in the Mekong Delta of Vietnam
Source: Trop Anim Health Prod. 2019 Jul 2;52(1):53–62. doi: 10.1007/s11250-019-01982-3 (PMC6969868; doi:10.1007/s11250-019-01982-3)
Supplement: Supplementary file 2 — (DOCX 15 kb) [file 11250_2019_1982_MOESM2_ESM.docx]

Table S1. Dimensions and mass of corresponding to 1 worm of each helminth species.

| Helminth species | *L* (*µ*m) | *D* (*µ*m) | *w* (mg) | Reference |
| --- | --- | --- | --- | --- |
| *Ascaridia galli* | 69,000 | 1,100 | 52.20 | doi: 10.4172/2332-2608.1000159 |
| *Heterakis gallinarum* | 7,000 | 280 | 0.34 | doi:10.4172/2332-2608.1000159 |
| *Cheilospirura hamulosa* | 14,850 | 345 | 1.10 | doi: 10.1155/2015/569340 |
| *Raillietina tetragona* | 250,000 | 3,000 | 1,406.3 | https://parasitipedia.net/index.php?option=com_content&view=article&id=2588&Itemid=2870 |
| *Raillietina cesticillus* | 130,000 | 2,000 | 325.0 | https://parasitipedia.net/index.php?option=com_content&view=article&id=2588&Itemid=2870 |
| *Raillietina echinobothrida* | 250,000 | 3,000 | 1,406.3 | https://parasitipedia.net/index.php?option=com_content&view=article&id=2588&Itemid=2870 |
| *Hymenolepis* spp*.* | 20,000 | 300 | 1.12 | The Chicken Health Handbook, 2^nd^ Edition: A Complete Guide to Maximizing Flock Health and Dealing with Disease |
| Echinostomatidae | 16,000 | 2,250 | 50.63 | http://www.fao.org/docrep/018/x0583e/x0583e.pdf |

*L*=body length (*μ*m); *D*=body diameter (*μ*m); *w*=Estimated weight of an individual helminth worm;
