# Supplementary material for: Characterisation of gastrointestinal helminths and their impact in commercial small-scale chicken flocks in the Mekong Delta of Vietnam
Source: Trop Anim Health Prod. 2019 Jul 2;52(1):53–62. doi: 10.1007/s11250-019-01982-3 (PMC6969868; doi:10.1007/s11250-019-01982-3)
Supplement: Supplementary file 4 — (DOCX 16 kb) [file 11250_2019_1982_MOESM4_ESM.docx]

Table S3. Agreement between the detection of different types of helminth detected in individual birds.

|  | Helminth detection result | | | | Kappa value  (SE) | p-value | Level of agreement |
| --- | --- | --- | --- | --- | --- | --- | --- |
|  | (1+)(2+) | (1+)(2-) | (1-)(2+) | (1-)(2-) |  |  |  |
| *Healthy birds* |  |  |  |  |  |  |  |
| (1) AG (2) HG | 21 | 11 | 31 | 57 | 0.254 (0.085) | 0.001 | Fair |
| (1) AG (2) cestodes | 12 | 29 | 6 | 82 | 0.356 (0.086) | 0.001 | Fair |
| (1) HG (2) cestodes | 15 | 37 | 3 | 65 | 0.275 (0.071) | <0.001 | Fair |
| *Diseased birds* |  |  |  |  |  |  |  |
| (1) AG (2) HG | 26 | 11 | 12 | 43 | 0.482 (0.104) | <0.001 | Moderate |
| (1) AG (2) cestodes | 53 | 25 | 2 | 12 | 0.321 (0.085) | <0.001 | Fair |
| (1) HG (2) cestodes | 53 | 25 | 1 | 13 | 0.357 (0.083) | <0.001 | Fair |

AG=*A. galli*; HG=*H. gallinarum*; SE=Standard Error
