# Supplementary material for: Characterisation of gastrointestinal helminths and their impact in commercial small-scale chicken flocks in the Mekong Delta of Vietnam
Source: Trop Anim Health Prod. 2019 Jul 2;52(1):53–62. doi: 10.1007/s11250-019-01982-3 (PMC6969868; doi:10.1007/s11250-019-01982-3)
Supplement: Supplementary file 5 — (DOCX 15 kb) [file 11250_2019_1982_MOESM5_ESM.docx]

Table S4: Linear regression models investigating variables associated with the number of helminth worms (log) in normal chicken flocks.

|  | Model 1  (Outcome=*A. galli*) | | Model 2  (Outcome=*H. gallinarum*) | | Model 3  (Outcome= Cestodes) | |
| --- | --- | --- | --- | --- | --- | --- |
|  | Univariable | Multivariable | Univariable | Multivariable | Univariable | Multivariable |
| Farmer’s age (years) (log) | -0.32 (0.29) | - | -0.19 (0.75) | - | 0.36 (0.11) |  |
| Gender (female) | 0.57 (0.02) | - | -0.36 (0.49) | - | 0.30 (0.11) |  |
| Length of cycle (log) | 1.08 (0.07) | - | 1.94 (0.10) | - | 0.47 (0.31) |  |
| House floor (Ref. Non-cement) |  |  |  |  |  |  |
| Cement | 0.41 (0.05) | 0.36 (0.09) | 0.38 (0.37) | - | 0.35 (0.03) | 0.42 (0.01) |
| Density (chickens/m^2^) (log) | 0.05 (0.66) | - | 0.13 (0.56) | - | -0.15 (0.08) |  |
| Ducks in farm | 0.08 (0.66) | - | -0.61 (0.08) | -0.72 (0.029) | -0.25 (0.06) | -0.30 (0.02) |
| Season (Ref. Dry) | 0.38 (0.02) | 0.34 (0.04) | 1.05 (<0.001) | 1.11 (<0.001) | 0.11 (0.39) |  |
| Anthelmintic used (Ref. Never) |  |  |  |  |  |  |
| Used >7 weeks before slaughter | 0.07 (0.23) | - | 0.35 (0.46) | - | -0.06 (0.18) | - |
| Used 7 weeks before slaughter | 0.40 (0.24) | - | 0.43 (0.47) | - | -0.12 (0.18) | - |
